# Prevalence of HIV-infection in Saudi Arabia

**DOI:** 10.1186/1753-6561-5-S6-P252

**Published:** 2011-06-29

**Authors:** AF Alothman, K Mohajer, H Balkhy

**Affiliations:** 1 Medicine Department, King Abdulaziz Medical City, Riyadh, Saudi Arabia; 2 College of Medicine, KSAU-HS, Riyadh, Saudi Arabia; 3 Infection Prevention and Control, King Abdulaziz Medical City, Riyadh, Saudi Arabia

## Introduction / objectives

Human immunodeficiency virus infection prevalence in Saudi Arabia was shown to be 0.02% in previous study. Following the annual incidence of HIV-infection can evaluate the degree of change in incidence of infection.

## Methods

Obtaining the correct data from the National AIDS Program at Saudi Ministry of Health according to the annual report of HIV-positive patients.

## Results

Between 1984 and 2001, it was found that 1285 HIV-positive cases were Saudis averaging 76 new cases per year. The reported HIV-positive person at the end of 2004 was 2005 cases. The rate of annual incidence of HIV-infection in Saudis was ranging 229-505 cases per year on 2001 - 2009. Between 2001 and 2009 the mean annual incidence of HIV-infection in Saudis was 342 cases per year. The last 8 years the new HIV infection in Saudis was 2734 cases only between early 2002 and end of 2009. The total number of HIV-positive Saudis on early 2010 was 4019 persons.

## Conclusion

During the last 8 years showed significant annual increase in the number of HIV-infected people in Saudi Arabia. This cumulative increase will require better policies to deal with such rising incidence of HIV-infection.

## Disclosure of interest

None declared.

